# The Arrows and Colors Cognitive Test (ACCT): A new verbal-motor free cognitive measure for executive functions in ALS

**DOI:** 10.1371/journal.pone.0200953

**Published:** 2018-08-09

**Authors:** Barbara Poletti, Laura Carelli, Andrea Faini, Federica Solca, Paolo Meriggi, Annalisa Lafronza, Luciana Ciringione, Elisa Pedroli, Nicola Ticozzi, Andrea Ciammola, Pietro Cipresso, Giuseppe Riva, Vincenzo Silani

**Affiliations:** 1 Department of Neurology and Laboratory of Neuroscience—IRCCS Istituto Auxologico Italiano, Milan, Italy; 2 Department of Cardiovascular, Neural and Metabolic Sciences—IRCCS Istituto Auxologico Italiano, Milan, Italy; 3 Department of Pathophysiology and Transplantation, “Dino Ferrari” Center, Università degli Studi di Milano, Milan, Italy; 4 ICT & Biomedical Technology Integration Unit, Centre for Innovation and Technology Transfer (CITT), Fondazione Don Carlo Gnocchi Onlus, Milan, Italy; 5 Department of Psychology and Cognitive Science, University of Trento, Rovereto, Italy; 6 Applied Technology for Neuro-Psychology Lab, IRCCS Istituto Auxologico Italiano, Milan, Italy; 7 Department of Psychology, Università Cattolica del Sacro Cuore, Milan, Italy; University of Toronto, CANADA

## Abstract

**Background and objective:**

The presence of executive deficits in patients with Amyotrophic Lateral Sclerosis is well established, even if standardized measures are difficult to obtain due to progressive physical disability of the patients. We present clinical data concerning a newly developed measure of cognitive flexibility, administered by means of Eye-Tracking (ET) technology in order to bypass verbal-motor limitations.

**Methods:**

21 ALS patients and 21 age-and education-matched healthy subjects participated in an ET-based cognitive assessment, including a newly developed test of cognitive flexibility (Arrows and Colors Cognitive Test–ACCT) and other oculomotor-driven measures of cognitive functions. A standard screening of frontal and working memory abilities and global cognitive efficiency was administered to all subjects, in addition to a psychological self-rated assessment. For ALS patients, a clinical examination was also performed.

**Results:**

ACCT successfully discriminated between patients and healthy controls, mainly concerning execution times obtained at different subtests. A qualitative analysis performed on error distributions in patients highlighted a lower prevalence of perseverative errors, with respect to other type of errors. Correlations between ACCT and other ET-based frontal-executive measures were significant and involved different frontal sub-domains. Limited correlations were observed between ACCT and standard ‘paper and pencil’ cognitive tests.

**Conclusions:**

The newly developed ET-based measure of cognitive flexibility could be a useful tool to detect slight frontal impairments in non-demented ALS patients by bypassing verbal-motor limitations through the oculomotor-driven administration. The findings reported in the present study represent the first contribution towards the development of a full verbal-motor free executive test for ALS patients.

## Introduction

Cognitive and behavioral changes in patients with Amyotrophic Lateral Sclerosis (ALS) are now recognized as integral features of the disease [1]. Despite recent findings showing the presence of heterogeneous cognitive profiles in ALS [2–4], the most commonly reported alterations involve executive functions, with literature consistently highlighting behavioral, neurophysiological, and cognitive correlates of frontal alterations in such populations [5,6]. The most consistent impairments have been observed on tests of phonemic verbal fluency [7] and remain evident even after the results were adjusted for verbal and motor impairment [8]. Verbal fluency execution involves processes of set-shifting, inhibition, sustained attention, and working memory [9]. A recent study highlighted that set-shifting and initiation are the most common executive sub-functions that are impaired in non-demented ALS patients; conversely, inhibition and problem-solving abilities seem less affected in this population [10]. Another study, involving meta-analysis, confirmed that set-shifting assessment performed by means of Wisconsin Card Sorting Test (WCST) [11] allows to detect a typical and selective component of executive functions impairment in ALS [12]. Other tests to assess global cognitive [Montreal Cognitive Assessment (MoCA)] and executive function [Frontal Assessment Battery (FAB) and Edinburgh Cognitive and Behavioural ALS Screen (ECAS)] failed to distinguish between patients and healthy controls.

In addition to set shifting abilities, cognitive flexibility is impaired in ALS patients due to limitations in executive control [13]. However, such observations have been collected by means of tests that did not accommodate for verbal and motor disability of ALS patients; such issues may at least partially account for the inconsistency of findings obtained by previous studies [14].

Executive dysfunction in ALS is recognized as a relevant factor that influences disease management and progression [15–17]. Moreover, longitudinal assessment of such abilities is helpful to clarify clinical phenotypes, according to biological and behavioral characteristics, and define prognosis. For these reasons, the development of validated motor-verbal free measures to assess cognitive functions, and in particular frontal-executive abilities, could improve both the phenotype description and care of patients [13].

Recently, some attempts have been made in order to obtain verbal-motor free indicators of changes in executive function of patients with ALS. In particular, event-related potentials (ERP) have been employed to assess frontal involvement with minimal motor demands [12, 14, 18–19]. ERP measurements require complex, expensive equipment and specific competencies in order to detect and analyze subtle changes in frontal cognitive activity in ALS patients without dementia. Moreover, the quantitative and qualitative data derived from ERP measurements are not comparable with scores obtained from standard cognitive testing. Therefore, these measures cannot be used for a longitudinal evaluation of neuropsychological functions. Recent studies have also used untimed measures that did not depend on a specific response modality, allowing patients to respond verbally or manually [20–22]. However, even the presence of minimal motor functions could be prevented in the advanced stages of the disease.

Eye Tracking (ET), a well-known and consistently validated Alternative and Augmentative Communication system, has been used with the aim of administering neuropsychological tests in a motor-verbal free manner [23–26]. We recently presented preliminary results showing an extensive neuropsychological battery developed for ET control, covering language, attentional and executive functions, and social cognition domains [27–29]. The current study describes clinical data on a newly developed measure of cognitive flexibility, the Arrows and Colors Cognitive Test (ACCT), administered by means of ET technology. Relationships between other validated standard measures and with the recently proposed oculomotor-driven neuropsychological battery are discussed, together with sensitivity in discriminating between ALS patients and healthy controls.

## Materials and methods

### Participants

Twenty-one ALS patients were recruited at the inpatient-outpatient ALS Center at the Department of Neurology of the IRCCS Istituto Auxologico Italiano, Milan. The diagnosis of definite ALS was made by an expert consultant neurologist, according to El Escorial Criteria [30]. Patients were excluded if they were in terminal stages of disease or had major medical, neurological, psychiatric history, and/or cardiovascular comorbidities. None of the patients had major visual impairments or oculomotor dysfunction. Twenty-one healthy subjects, matched for age and educational levels, were also recruited. All participants were native Italian speakers.

For ALS patients, clinical and laboratory assessments included evaluation of disability, using the revised ALS Functional Rating Scale (ALSFRS/R) [31], and of respiratory function with spirometry.

All participants completed the designed cognitive and psychological protocols and the ET-based assessment, as described below.

The study protocol was reviewed and approved by the Ethics Committee of IRCCS Istituto Auxologico Italiano (ethics committee code: 2011_04_12_16) and all eligible subjects received verbal and written information about the study. All participants signed an informed consent, according to the Declaration of Helsinki. The individual in this manuscript (S1 and S2 Videos) has given written informed consent (as outlined in PLOS consent form) to publish these case details.

### Cognitive and psychological assessment

#### ACCT: ET-based assessment of cognitive flexibility

**Fig 1:**
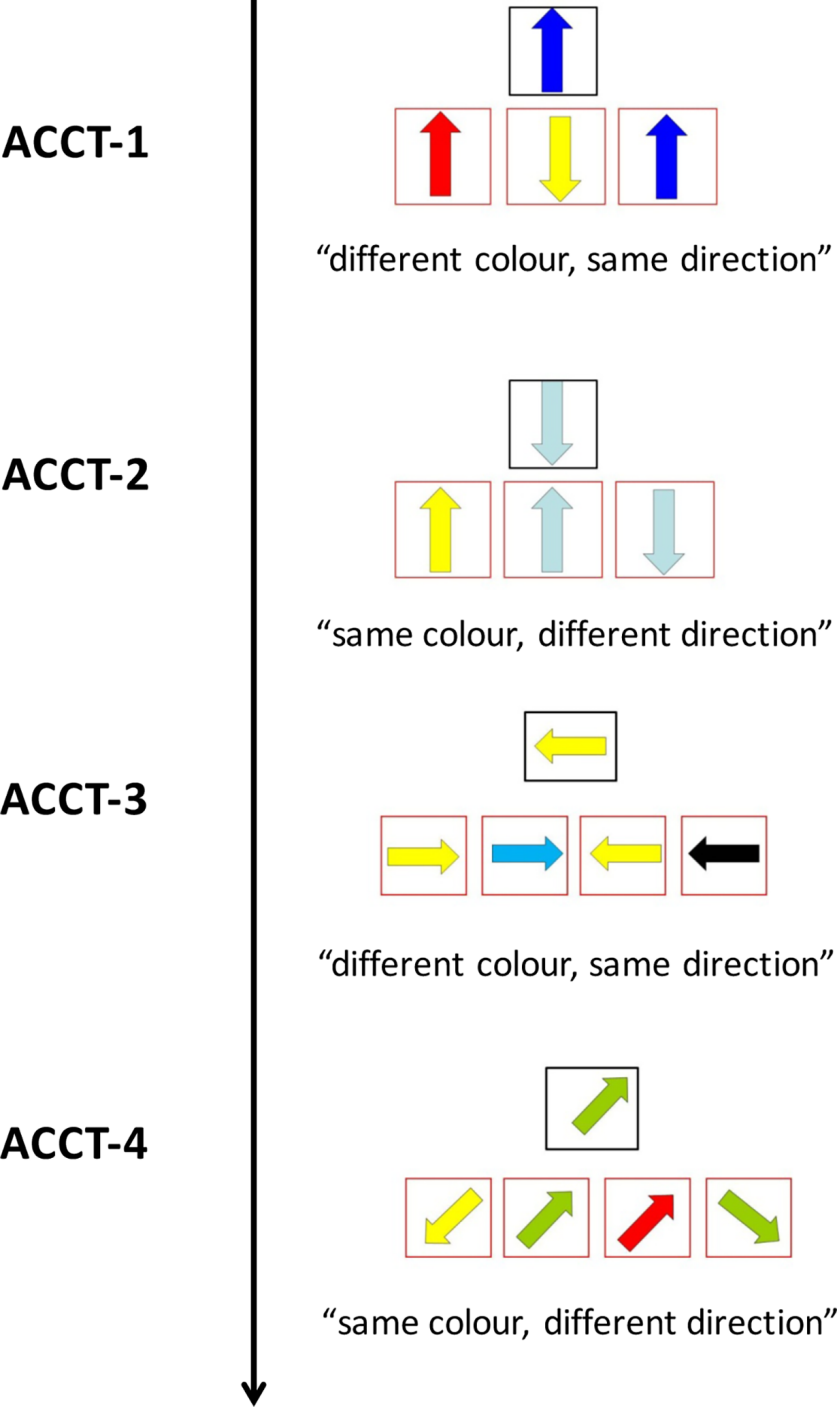
Structure and instructions of the four ACCT subtests.

ACCT is composed by four rows (ACCT-1, ACCT-2, ACCT-3, ACCT-4), made of 12 items each, where participants are required to select the appropriate arrow according to a written instruction. The patients were allowed to practice each trial twice before the start of data collection. Two types of instructions were presented. In the first (ACCT-1, see S1 Video; the individual in this manuscript has given written informed consent, as outlined in PLOS consent form, to publish these case details) and third (ACCT-3) trials: “*Note the arrow in the upper center*. *In the following exercise*, *you will be asked to choose between the arrows below*, *the one with the different color*, *but the same direction as that of the target arrow*”. In the second (ACCT-2) and fourth (ACCT-4, see S2 Video; the individual in this manuscript has given written informed consent, as outlined in PLOS consent form, to publish these case details) trials: “*Note the arrow in the upper center*. *In the following exercise*, *you will be asked to choose*, *between the arrows below*, *the one with the same color*, *but a different direction from that of the target arrow*”. Upper arrows are equally distributed between four directions (3 arrows for each direction). Target arrows are equally distributed between the right, left and central positions (4 for each position in 3-arrow trials, 3 for each position in 4-arrow trials). For each task, two or three distracters were present for ACCT-1 and ACCT-2 and for ACCT-3 and ACCT-4 respectively; distracters were represented by items following a part of the instruction (i.e. color or direction), and by a perseverative response over the previous subtest (see Fig 1). The mean latency, mean latency standard deviation, and the total number of correct responses were recorded for each subtest. Moreover, partial errors (i.e. following a part of the instructions) and perseverative errors were computed for each subtest, in order to obtain additional qualitative data about subjects’ performances.

#### Other standard and ET-based cognitive and psychological tests

A neuropsychological and psychological protocols employed in previous studies were adopted, including both standard and ET-based cognitive measures. In particular, standard cognitive measures included two brief batteries, for dysexecutive syndrome, FAB [32], and global cognitive functioning, MoCA [33], respectively, and a working memory—WM subtest, Digit Sequencing Task of the Brief Assessment of Cognition in Schizophrenia (BACS) [34].

In addition, a battery of neuropsychological tests, previously adapted to oculomotor control by means of ET technology, was employed for evaluating language (Token Test), attentional abilities (d2 Test), executive functions (Raven’s Colored Progressive Matrices—RCPM, Modified Card Sorting Test–MCST, Iowa Gambling task–IGT) and social cognition (Reading the Mind in the eyes test–RME) [28,29].

In addition, participants completed a screening for symptoms of depression and anxiety, using both the Beck Depression Inventory (BDI) and the State-Trait Anxiety Inventory-Y (STAI-Y). BDI was used to assesses both cognitive-affective (BDI-CA) and somatic-performance (BDI-SP) symptoms of depression [35], and STAI-Y evaluated both state anxiety level (STAI-Y1) and trait anxiety (STAI-Y2) components [36]. For ALS patients, a behavioral assessment was also performed using the Frontal Behavioral Inventory (FBI) [37].

### Eye-Tracking

An EyeLink 1000 infrared tracker was used to record participants’ eye movements throughout all the experimental conditions. Head position was stabilized using a chin rest, at a distance from the screen of around 70 cm. A nine-points calibration covering the totality of the visual screen and a drift correction were carried out before each test, in order to ensure an accurate eye position recording. In order to select an item on the computer screen, participants were required to fixate on the item for a minimum of 80% over a period of 1,500 milliseconds. Eye movement data consisted of moment-to-moment measures of eyes' displacements along the vertical and horizontal axes (in millimeters) within the spatial working area of the monitor screen (resolution 1024 x 768 pixels). Pupil dilation and gazes were acquired, based on pupil position and corneal reflection on the frontal surface of participants’ eyes (caused by an infrared light source), at 250 Hz by means of an EyeLink 1000 system using a new software suite, named the eBrain Test Engine (ETE, http://ebrainengine.codeplex.com/). Acquired signals were also analyzed using the following off-line tools: Experiment Builder (SR Research, Ottawa, Canada), Excel (Microsoft, Redmond, USA) and custom programs written in MATLAB 7.2 (The Mathworks, Inc.; Natick, MA). A training phase was performed, as described in previous works [28,29].

### Procedure

The overall experimental procedure was performed along two sessions within a one week period. In the first session, standard cognitive tests and psychological measures were administered. In addition, the subjects participated in a training phase for the ET evaluation. During the second session, participants underwent the ET-based neuropsychological assessment. The administration order of each ET neuropsychological test was randomized among subjects, in order to avoid practice and fatigue effects.

### Statistical analysis

Descriptive statistics (mean ± standard deviations for continuous variables and frequencies for discrete variables) were used to describe the main characteristics of the recruited samples and performances of the ET and standard cognitive/psychological assessments. Distribution of the variables in terms of proximity to normal curve and the homogeneity of variances were detected by the Shapiro-Wilk test and Bartlett test, respectively. To compare the mean scores between groups, a two-sided t-test with pooled estimates of the sample variance or the Welch approximation were employed when the continuous variables were normally distributed, homoscedastic or heteroscedastic, respectively. Otherwise, a two-sided signed-rank test was performed. Finally, to assess the degree of association between scores in the ALS patients, the Spearman correlation coefficient was adopted. P-values were adjusted with for a false discovery rate.

To detect the sensitivity and the specificity of ET test, the area under the receiver operating characteristic (ROC) curve (AUC) was evaluated; cut off values were identified according to the Youden's J statistic method [38]. An α level of 0.05 was used for all hypothesis tests. All data analyses were performed using R Core Team (2014), Vienna, Austria.

## Results

### Healthy participants and patients’ demographic and clinical characteristics

Twenty-one ALS patients (males: 18; females: 3; age: 59.33 ± 11.84 years; education: 11.52 ± 3.39 years; disease duration: 33.62 ± 42.71 months) and 21 matched healthy participants (males: 9; females: 12; age: 57.38 ± 10.78 years; education: 12.52 ± 3.47) were recruited. For the patients’ group, clinical neurological examination showed an ALSFRS/R score of 37.57 ± 6.05 (ALSFRS/R Bulbar: 9.67 ± 2.89). Ten ALS patients had upper limb regions at onset, 8 had lower limb regions, 3 were bulbar, and no patient had respiratory symptoms at onset. According to the recent Strong and colleagues’ classification of ALS-FTD spectrum disorder (Strong et al., 2017), four patients (22.22%) were classified as having ALS with behavioral impairment (ALSbi) and two (12.5%) with cognitive impairment (ALSci). No patient was classified as having ALS which met the criteria for both ALSci and ALSbi (ALScbi), ALS with frontotemporal dementia (ALS-FTD) or ALS-dementia. At the standard neuropsychological assessment, patients performed significantly worse than healthy participants at the MoCA (ALS patients: 25.53 ± 2.43, controls: 27.14 ± 1.93, t = -2.28, p-value = .028), and the WM subtest (ALS patients: 20.19 ± 3.34, controls: 22.28 ± 2.34, Z = -2.22, p-value = .025), while no differences were observed with FAB (ALS patients: 16.30 ± 1.06, controls: 16.50 ± 1.18, t = -0.54, p-value = .60).

### ACCT results: Between groups differences

The performances of patients and controls were different when assessed through ACCT, with significantly higher values of mean latency observed for patients at ACCT-1, ACCT-2, and ACCT-4 subtests. Significantly higher values of the mean latency standard deviation were observed for patients for the ACCT-1 and ACCT-4 assessments, suggesting a larger variability of performances in this group compared to the control group. A tendency towards a significant difference in number of correct responses was observed for ACCT-4, with lower scores for patients (Table 1). Significantly higher values of mean latency for only correct responses were observed in ALS patients’ group, for ACCT-1, ACCT-2, and ACCT-4 subtests (S1 Table).

**Table 1:** Performance on ACCT subtests in ALS patients and healthy subjects. Data are expressed as Means (SD).

	ALS patients N = 21	Healthy controls N = 21		
	Mean (SD)	Mean (SD)	*W/Z*	*p-value*
ACCT-1 *mean latency*	3.804 (0.798)	3.253 (0.452)	**317**	.015
ACCT-2 *mean latency*	4.288 (0.970)	3.647 (0.657)	**312**	.021
ACCT-3 *mean latency*	4.356 (0.826)	3.976 (0.770)	287	.1
ACCT-4 *mean latency*	4.282 (1.088)	3.503 (0.650)	**318**	.014
ACCT-1 *sd latency*	1.215 (1.680)	0.538 (0.403)	**311**	.023
ACCT-2 *sd latency*	1.204 (0.790)	0.798 (0.568)	292	.07
ACCT-3 *sd latency*	1.167 (0.534)	0.855 (0.366)	293	.07
ACCT-4 *sd latency*	1.617 (1.156)	0.855 (0.523)	**313**	.020
ACCT-1 *n° correct*	11.81 (0.51)	11.90 (0.30)	-0.515	.7
ACCT-2 *n° correct*	10.86 (2.65)	11.71 (0.46)	-1.417	.2
ACCT-3 *n° correct*	10.62 (2.50)	11.57 (0.81)	-1.685	.1
ACCT-4 *n° correct*	10.71 (2.72)	11.57 (1.08)	-1.838	.07

Bold numbers indicate statistical significance with *p* < 0.05.

Abbreviations: W = Wilcoxon; Z = exact Wilcoxon Mann-Whitney test; sd latency = mean latency standard deviation; n° correct = number of correct responses.

The ROC curve analysis supported the sensitivity of ACCT-1, ACCT-2, ACCT-4 mean latency and mean latency standard deviation variables (AUC values >0.70), according to the determined cut-off scores. AUC values between 0.6 and 0.7 were found for ACCT-3 number of correct responses and for ACCT-3 mean latency.

**Fig 2:**
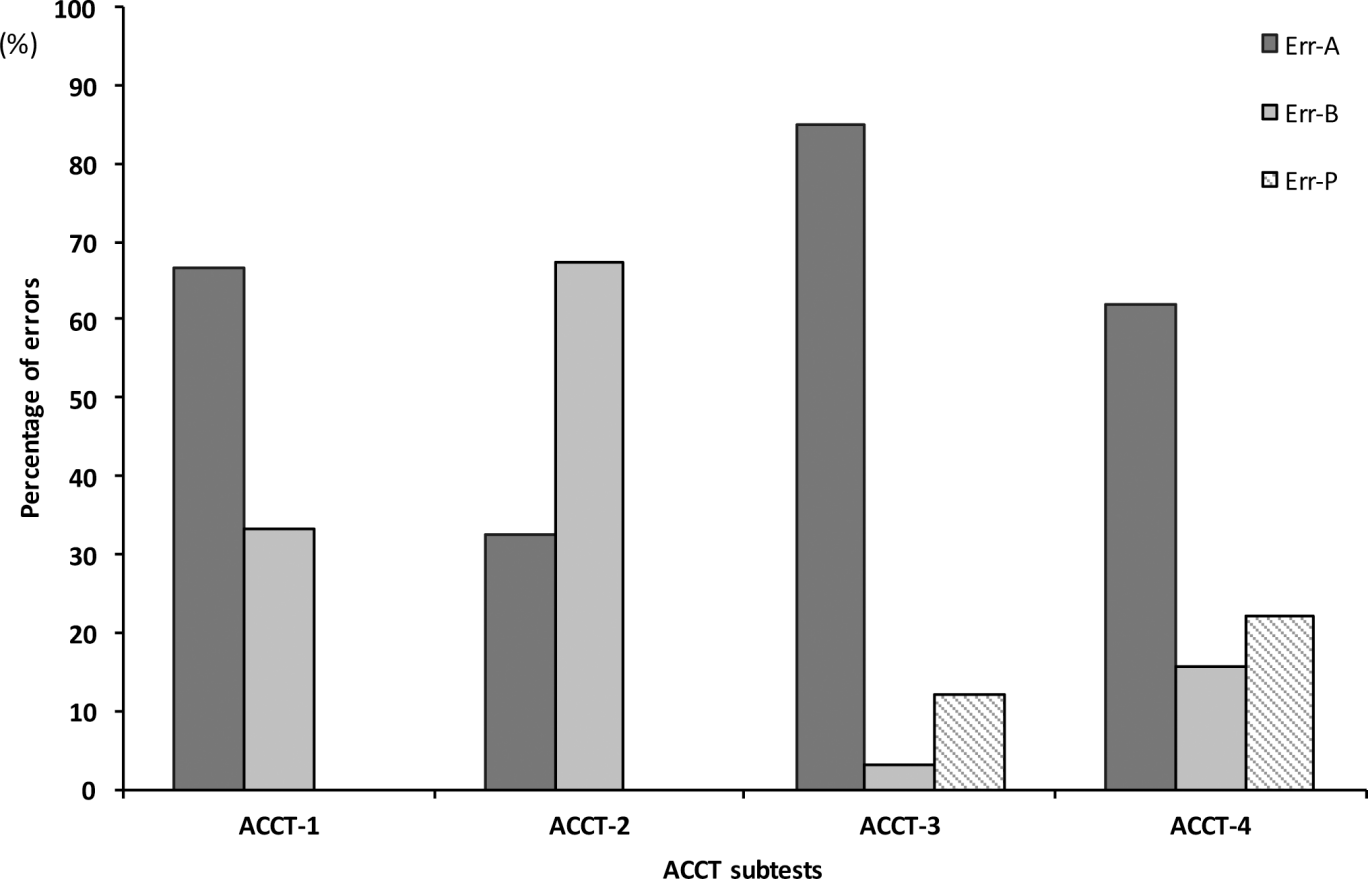
Errors distribution at the ACCT subtests in ALS patients. Err-A = partial errors following the ‘colour’ rule; Err-B = partial errors following the ‘direction’ rule; Err-P = perseverative errors.

A qualitative analysis on patients’ performances of the types of errors committed highlighted an increasing number of perseverative errors during the ACCT-3 to ACCT-4 subtests. However, perseverative errors were globally less present than partial ones, with a prevalence of partial (color) errors in ACCT-1, ACCT-3 and ACCT-4 subtests. For ACCT-2, a higher proportion of partial (direction) errors, with respect to partial (color) ones, was observed (Fig 2).

### ACCT results: Correlation with other standard and ET-based cognitive tests

When considering the ALS group (Table 2), significant correlations were observed between MoCA and ACCT-1 mean latency and number of correct responses; between FAB and ACCT-3 number of correct responses. Correlations between ACCT and WM subtest only concerned mean latency standard deviation of ACCT-2, even if a tendency toward a significant correlation (p-value = .06) was observed for the mean latency standard deviation of ACCT-3, and number of correct responses for ACCT-2. For controls (Table 2), significant correlations were observed between MoCA and the number of correct responses of ACCT-3, with a mean latency trend close to significant (p-value = .06) for ACCT-3; between WM subtest and the mean latency standard deviation of ACCT-2, with a trend toward a significant correlation (p-value = .06) for mean latency correlation of ACCT-2. Conversely, the FAB did not correlated with any of the ACCT variables in controls’ groups.

**Table 2:** Correlations between ACCT subtests and standard tests in patients and controls.

	ALS patients			Healthy controls		
	MoCA	WM	FAB	MoCA	WM	FAB
ACCT-1 *mean latency* (p-value)	-.48 (.05)	-.43 (.2)	-.06 (.8)	-.34 (.1)	.02 (.9)	-.24 (.3)
ACCT-2 *mean latency* (p-value)	-.34 (.2)	-.44 (.2)	-.05 (.8)	-.38 (.09)	-.49 (.06)	-.03 (.9)
ACCT-3 *mean latency* (p-value)	-.05 (.9)	-.42 (.2)	-.08 (.7)	-.42 (.06)	-.35 (.2)	.12 (.6)
ACCT-4 *mean latency* (p-value)	-.11 (.7)	-.43 (.2)	-.10 (.7)	.06 (.8)	.17 (.5)	-.21 (.4)
ACCT-1 *sd latency* (p-value)	-.27 (.3)	-.15 (.6)	.16 (.5)	-.33 (.1)	-.14 (.6)	-.16 (.5)
ACCT-2 s*d latency* (p-value)	-.24 (.4)	**-.60** (.038)	.13 (.6)	-.28 (.2)	**-.59** (.020)	.00 (>.9)
ACCT-3 *sd latency* (p-value)	-.28 (.3)	-.55 (.06)	.05 (.8)	-.27 (.2)	-.28 (.3)	.04 (.9)
ACCT-4 *sd latency* (p-value)	-.32 (.2)	-.29 (.4)	-.27 (.3)	.03 (>.9)	.01 (>.9)	-.27 (.2)
ACCT-1 *n° correct* (p-value)	**.49** (.044)	-.32 (.3)	.06 (.8)	.29 (.2)	.39 (.2)	-.12 (.6)
ACCT-2 *n° correct* (p-value)	.33 (.2)	.56 (.06)	.08 (.8)	-.26 (.3)	-.19 (.5)	.19 (.4)
ACCT-3 *n° correct* (p-value)	.13 (.6)	.22 (.5)	**-.53** (.025)	**.49** (.023)	.35 (.2)	-.17 (.5)
ACCT-4 *n° correct* (p-value)	.30 (.2)	.00 (>.9)	.08 (.7)	.10 (.7)	-.11 (.7)	-.00 (>.9)

Bold numbers indicate statistical significance with *p* < 0.05.

Abbreviations: MoCA = Montreal Cognitive Assessment; WM = Working Memory subtest; FAB = Frontal Assessment Battery; sd latency = mean latency standard deviation; n° correct = number of correct responses.

When correlations between ACCT and other ET-based neuropsychological tests were considered, the ALS group showed associations between mean latency and mean latency standard deviation of the ACCT subtests and frontal-executive measures (d2, MCST, RCPM, IGT), together with RME as a measure of social cognition (Table 3 and S2 Table). Response accuracy of the ACCT-2 subtest correlated with RME (number of correct responses) (S2 Table). For controls, correlations were less extended and mainly regarded mean latency and mean latency standard deviation of ACCT-2 and ACCT-3 subtests that correlated with frontal-executive measures and control task at the RME (Table 3 and S3 Table). In this group, response accuracy for the ACCT-1 subtest correlated with d2, MCST and RCPM measures; some trends were also observed between response accuracy for the ACCT-3 subtest and the RCPM and RME control task (S3 Table).

**Table 3:** Correlations between ACCT subtests and other ET-based tests in patients and controls.

	ALS patients				Healthy controls			
	ACCT-1 *Mean latency*	ACCT-2 *Mean latency*	ACCT-3 *Meanlatency*	ACCT-4 *Mean latency*	ACCT-1 *Mean latency*	ACCT-2 *Mean latency*	ACCT-3 *Mean latency*	ACCT-4*Mean latency*
d2 *mean latency* (p-value)	**.73** (< .001)	**.68** (.002)	.45 (.06)	**.49** (.033)	.40 (.07)	.18 (.4)	**.50** (.020)	.08 (.7)
d2 *n° correct* (p-value)	**-.46** (.046)	-.28 (.3)	**-.46** (.045)	-.14 (.6)	-.18 (.4)	-.33 (.1)	-.28 (.2)	-.17 (.5)
MCST *mean latency* (p-value)	**.55** (.017)	.32 (.2)	**.46** (.048)	**.46** (.046)	.35 (.1)	.15 (.5)	**.56** (.009)	.07 (.8)
MCST *n° correct* (p-value)	-.19 (.4)	-.27 (.3)	-.42 (.08)	**-.47** (.044)	-.31 (.2)	-.003 (>.9)	-.43 (.050)	-.08 (.7)
MCST *n° categories* (p-value)	-.16 (.5)	-.37 (.1)	-.44 (.053)	-.40 (.08)	-.32 (.2)	.002 (>.9)	-.43 (.050)	-.08 (.7)
MCST *n° cards* (p-value)	.19 (.4)	.37 (.1)	**.47** (.038)	**.47** (.037)	.30 (.2)	.003 (>.9)	38 (.09)	-.15 (.5)
RCPM *mean latency* (p-value)	.44 .06)	.40 .09)	**.57** .010)	**.48** .037)	.26 .3)	.42 .06)	**.74** < .001)	.35 .1)
RCPM *n° correct* (p-value)	**-.53** (.018)	-.26 (.3)	-.25 (.3)	**-.49** (.034)	-.27 (.2)	**-.53** (.012)	-.39 (.08)	-.15 (.5)
RME *mean latency* (p-value)	.28 .2)	.12 .6)	**.49** .036)	-.05 .9)	.04 .9)	.26 .3)	.18 .4)	.16 .5)
RME *n° correct* (p-value)	-.31 (.2)	-.18 (.5)	.06 (.8)	-.20 (.4)	-.42 (.06)	-.23 (.3)	-.10 (.7)	.14 (.6)
RMEc *mean latency* (p-value)	**.84** (< .001)	**.62** (.005)	**.47** (.043)	.43 (.07)	.38 (.09)	**.53** (.015)	**.56** (.009)	.22 (.3)
RMEc *n° correct* (p-value)	-.39 (.1)	-.24 (.3)	-.21 (.4)	-.27 (.3)	-.13 (.6)	-.24 (.3)	-.23 (.3)	.07 (.8)
IGT *mean latency* (p-value)	**.67** (.002)	**.57** (.012)	**.72** (< .001)	**.62** (.006)	.21 (.4)	.42 (.06)	.36 (.1)	.33 (.1)

Bold numbers indicate statistical significance with *p* < 0.05.

Abbreviations: n° correct = number of correct responses; n° categories = number of achieved categories; n° cards = number of used cards; RMEc = Reading the Mind in the eyes test–control test.

### ACCT results: Correlation with clinical and psychological parameters

No significant correlations were observed between ACCT variables (mean latency and number of correct responses), and either the disease onset or ALSFRS/R in the ALS patients’ group.

With regard to psychological components in the patients’ group, a negative correlation was observed between state anxiety level (STAI-Y1) and mean latency standard deviation for the ACCT-2 (rho = -.47; p-value = .031) and ACCT-3 (rho = -.59; p-value = .006) subtasks. Trait anxiety (STAI-Y2) was positively correlated in this sample with number of correct responses at the ACCT-3 subtask (rho = .45; p-value = .043).

The trait anxiety (STAI-Y2) for the control group was positively correlated with mean latency at both ACCT-1 (rho = .52; p-value. = .016), ACCT-2 (rho = .52; p-value = .016) and ACCT-3 (rho = .46; p-value = .035) subtests. Moreover, the level of depression (BDI) was positively correlated with the mean latency for ACCT-1 (BDI-SP: rho = .44; p-value = .043) and ACCT-2 subtests (BDI-TOT tot: rho = .53; p-value = .013; BDI-CA: rho = .43; p-value = .05; BDI-SP: rho = .60; p-value = .004), and with the mean latency standard deviation for ACCT-2 (BDI-TOT: rho = .46; p-value = .036; BDI-CA: rho = .50; p-value = .022).

No significant correlations were observed between ACCT subtest variables (mean latency, mean latency standard deviation and number of correct responses) and patients’ behavioral examination (FBI).

## Discussion

The ACCT we presented, highlighted the decreased performances of time-related measures of ALS patients, when compared to controls. Less consistent results were observed with accuracy-related measures, i.e. number of correct responses, with a trend towards a significant difference observed across trials, from ACCT-1 to ACCT-4. ACCT-3 seems slightly more difficult than the other subtests, according to the observed mean values and the lack of differences between patients and controls performances. Maybe, the increase of arrows numerosity from ACCT-2 to ACCT-3 and the progressive instructions alternation involve a more significant working memory load, as also suggested by the appearance of perseverative errors.

Overall, our findings suggest the presence of slight cognitive changes concerning cognitive flexibility and set-shifting abilities in our non-demented ALS sample that may overtly affect execution times and, to a lesser extent, performance accuracy. Recent findings have highlighted functional reorganization in ALS patients during cognitive tasks as an early adaptive process to neuronal cell loss and time-related aspects could represent the initial clinical signs of cognitive change in our ALS population [39]. Longitudinal evaluation of patients’ performances using the ACCT test and other cognitive tasks could identify changes in performances across disease progression and better characterize cognitive changes. Furthermore, it is worth noting the few patients with ALSci in our sample (12.5%). Our small sample size could explain the limited presence of cognitive involvement detected by the ACCT.

In contrast with our results, in a recent study, Pettit and colleagues concluded that processing speed, when isolated from motor functions and high-order cognitive processes, is preserved in ALS patients, and, therefore, does not contribute to the frequently observed cognitive changes in such a population [9]. A possible explanation of these controversial findings could be that, in our study, the use of an oculomotor-driven version of the cognitive tests could affect performance speed, due to the possible presence of ocular motor disorders as a marker of sub-clinical frontal lobe dysfunction observed in ALS patients [40–42]. However, the clinical examination that preceded the administration of the experimental protocol excluded the presence of significant oculomotor dysfunctions. Moreover, the employed paradigm minimized the oculomotor component necessary to provide responses to the presented items. Finally, no correlation between patients’ physical function decline (ALSFRS/R) and mean latency at the ACCT subtests was observed. Similarly, disease onset did not correlate with time-related variables provided by the ACCT test. Overall, such results suggest that motor limitation severity and progression did not influence the speed of processing in our sample, and that such time-related aspects are likely related to an underlying cognitive involvement.

Correlations between ACCT and other ET-based measures of frontal-executive abilities were extended and congruent, and mainly involved the mean latency variables of ACCT. Some relationships have also been observed between ACCT and a measure of social cognition, RME, suggesting a role for set-shifting and frontal-executive abilities in such function, as reported previously [4].

Otherwise, ACCT demonstrated limited correlation with other standard cognitive tests, such as the MoCA and a WM measure (Digit Sequencing Task), and very poor correlation with a widely used battery that assesses frontal abilities (FAB). Discrepancy between the FAB and ACCT tests could be explained by the poor sensitivity of the ‘paper and pencil’ frontal battery in non-demented ALS patients, as suggested by recent literature [43] and confirmed by our study. Moreover, it is also likely that FAB and ACCT assessments target different components of executive function. With regard to WM subtest, although several studies have proposed a relationship between frontal-executive and WM abilities [44–46] and mild dysfunction of WM abilities are consistent with previous, multidimensional reports [5, 47], we observed limited correlation in our study between ACCT performances and WM subtests. Further refinements of the developed test, aimed at increasing the level of difficulty and the sensitivity of the different subtests, as well as comparison with other WM assessment tools, could provide more consistent data.

In addition to time and accuracy-related variables, the developed ACCT test allows the collection of more qualitative data about frontal-executive involvement than other available tests, according to the categorization of errors presented. The preliminary findings presented here suggest a reduced presence of perseverative errors, with respect to other type of errors, in our sample of non-demented ALS patients. Such results are in accordance with a previous study using a similar population where impaired inhibition was not a prominent feature [10]. Moreover, the use of a sophisticated and computerized tool, such as ET, could enable the collection of findings that would provide insight into the nature of errors that patients make when scanning items (i.e. perseverative, due to impulsivity or disinhibition). Further refinements of this protocol, including detection of oculomotor performances, and recruiting patients with more heterogeneous and severe cognitive impairments, could improve the test sensitivity and provide more subtle indications about cognitive performances.

## Conclusions

In ALS, frontal abilities, and particularly cognitive flexibility, are known to be impaired due to limitations in executive control in such patients. However, observations supporting such changes have been often collected by means of tests that do not accommodate for verbal and motor disabilities. The detection of cognitive impairments during the course of the disease is not fully and reliably accomplished by means of ‘paper and pencil’ tests, because the motor and verbal limitations are severe in moderate-severe stages of ALS. Such issues may at least partially account for the inconsistency of findings obtained by previous studies about specific components of cognitive involvement in the disease, including subcomponents of frontal-executive abilities.

We presented preliminary results about an extensive neuropsychological battery developed for oculo-motor control by means of ET, covering language, attentional and executive functions, and social cognition domains. The current study provides clinical data of a newly developed measure, the Arrows and Colors Cognitive Test (ACCT), administered by means of ET technology. The newly developed test allows a specific assessment of cognitive flexibility. Such measures, when integrated to other complementary tasks, could provide a more specific evaluation of different frontal changes involved in the course of ALS.

Our results propose the use of ACCT as a tool to assess cognition in patients with verbal-motor disabilities, such as ALS, when standard measures are not fully administrable. Further efforts will be aimed at investigating the feasibility of the developed system, together with validity and usability components, in larger populations of ALS patients. In particular, the inclusion of ALS patients presenting overt cognitive or behavioral impairments, or FTD, will be useful for improving ACCT clinical sensitivity. Finally, future research will be aimed at enhancing the ACCT number of items and level of difficulty in order to improve both its sensitivity and accuracy.

Despite these limitations, the findings reported in the present study represent the first contribution towards the development of a full verbal-motor free executive test for ALS patients. Accommodating or compensating for verbal-motor component is a crucial issue for longitudinal assessment of cognition in ALS. Adequately assessing such cognitive components could enhance clinical observations and better describe ALS patients’ phenotypes along the course of the disease, avoiding biases present in clinical and research settings.

## Supporting information

S1 Table Performance on ACCT subtests in ALS patients and healthy subjects, with concern to correct responses. Data are expressed as Means (SD).
(DOCX)

S2 Table Correlations between ACCT subtests and other ET-based tests in patients’ group. Bold numbers indicate statistical significance with p < 0.05.
(DOC)

S3 Table Correlations between ACCT subtests and other ET-based tests in healthy controls’ group. (DOC)

S1 Video Participant performing ACCT-1 subtest. (MOV)

S2 Video Participant performing ACCT-4 subtest. (MOV)
