# Supplementary material for: The Arrows and Colors Cognitive Test (ACCT): A new verbal-motor free cognitive measure for executive functions in ALS
Source: PLoS One. 2018 Aug 9;13(8):e0200953. doi: 10.1371/journal.pone.0200953 (PMC6084851; doi:10.1371/journal.pone.0200953)
Supplement: S1 Table — Data are expressed as Means (SD). (DOCX) [file pone.0200953.s001.docx]

|  | **ALS patients**  **N = 21** | **Healthy controls**  **N = 21** |  |  |
| --- | --- | --- | --- | --- |
|  | Mean (SD) | Mean (SD) | *W/Z* | *p-value* |
| ACCT-1 ‘*mean latency’* | 3.791 (0.788) | 3.262 (0.476) | **316** | .016 |
| ACCT-2 ‘*mean latency’* | 4.383 (1.048) | 3.654 (0.664) | **304** | .014 |
| ACCT-3 ‘*mean latency’* | 4.322 (0.839) | 3.985 (0.777) | 275 | .2 |
| ACCT-4 ‘*mean latency’* | 4.361 (1.276) | 3.476 (0.600) | **301** | .017 |
| ACCT-1 ‘*sd latency’* | 1.223 (1.835) | 0.540 (0.411) | **308** | .028 |
| ACCT-2 ‘*sd latency’* | 1.223 (0.808) | 0.788 (0.564) | 279 | .07 |
| ACCT-3 ‘*sd latency’* | 1.165 (0.578) | 0.841 (0.330) | 278 | .08 |
| ACCT-4 ‘*sd latency’* | 1.656 (1.409) | 0.844 (0.514) | 281 | .07 |

**S1 Table. Performance on ACCT subtests in ALS patients and healthy subjects, with concern to correct responses.**

Data are expressed as Means (SD).

Bold numbers indicate statistical significance with *p* < 0.05.

Abbreviations: W=Wilcoxon; Z= exact Wilcoxon Mann-Whitney test; sd latency= mean latency standard deviation.
