# Supplementary material for: The Arrows and Colors Cognitive Test (ACCT): A new verbal-motor free cognitive measure for executive functions in ALS
Source: PLoS One. 2018 Aug 9;13(8):e0200953. doi: 10.1371/journal.pone.0200953 (PMC6084851; doi:10.1371/journal.pone.0200953)
Supplement: S2 Table — Bold numbers indicate statistical significance with p < 0.05. (DOC) [file pone.0200953.s002.doc]

**S2 Table. Correlations between ACCT subtests and other ET-based tests in patients’ group**

|  | d2  *mean latency* | d2  *n°*  *correct* | MCST  *mean latency* | MCST  *n°*  *correct* | MCST  *n° categories* | MCST  *n°*  *cards* | RCPM  *mean latency* | RCPM  *n°*  *correct* | RME  *mean latency* | RME  *n°*  *correct* | RMEc  *mean latency* | RMEc  *n°*  *correct* | IGT  *mean latency* |
| --- | --- | --- | --- | --- | --- | --- | --- | --- | --- | --- | --- | --- | --- |
| ACCT-1 *sd latency*  p-value | **.52**  .024 | -.27  .3 | .34  .2 | -.03  .9 | .06  .8 | -.03  .9 | .29  .2 | **-.52**  .024 | .16  .5 | -.15  .6 | **.69**  .002 | -.43  .07 | **.48**  .037 |
| ACCT-2 *sd latency*  p-value | **.57**  .012 | **-.46**  .049 | .33  .2 | -.33  .2 | -.38  .1 | .28  .2 | **.48**  .037 | -.13  .6 | .13  .6 | -.05  .8 | .38  .1 | .02  >.9 | **.49**  .034 |
| ACCT -3 *sd latency*  p-value | .28  .2 | -.28  .2 | .42  .07 | **-.46**  .047 | **-.54**  .013 | **.56**  .010 | .54  **.016** | -.35  .1 | .35  .1 | -.07  .8 | **.46**  .049 | -.15  .5 | **.50**  .029 |
| ACCT-4 *sd latency*  p-value | **.51**  .028 | -.15  .5 | .46  .050 | -.33  .2 | -.29  .2 | **.46**  .039 | .32  .2 | **-.56**  .012 | -.30  .2 | -.32  .2 | .39  .1 | -.35  .1 | **.57**  .011 |
| ACCT-1  *n° correct*  p-value | -.24  .3 | .29  .2 | .09  .7 | -.17  .5 | -.16  .5 | -.03  .9 | -.08  .7 | .09  .7 | .09  .7 | .37  .1 | -.43  .06 | .26  .3 | -.19  .4 |
| ACCT-2  *n° correct*  p-value | -.41  .08 | -.02  >.9 | -.28  .3 | .05  .8 | .03  .9 | -.09  .7 | -.32  .2 | .23  .3 | -.06  .8 | **.69**  .001 | -.33  .2 | .16  .5 | -.20  .4 |
| ACCT-3  *n° correct*  p-value | .10  .7 | .05  .8 | -.01  >.9 | .11  .7 | .08  .7 | -.10  .7 | -.29  .2 | .14  .6 | -.32  .2 | .14  .6 | -.37  .1 | .17  .5 | -.16  .5 |
| ACCT-4  *n° correct*  p-value | -.08  .7 | -.14  .6 | -.14  .6 | .08  .7 | .10  .7 | -.27  .3 | -.16  .5 | .43  .07 | .27  .3 | .09  .7 | .13  .6 | .-14  .6 | .-27  .3 |

Bold numbers indicate statistical significance with *p* < 0.05.

Abbreviations: MCST= Modified Card Sorting test; RCPM= Raven’s Colored Progressive Matrices; RME= Reading the Mind in the eyes test; RMEc= Reading the Mind in the eyes test – control test; IGT= Iowa Gambling Task; sd latency= mean latency standard deviation; n° correct= number of correct responses.
