# Supplementary material for: The Arrows and Colors Cognitive Test (ACCT): A new verbal-motor free cognitive measure for executive functions in ALS
Source: PLoS One. 2018 Aug 9;13(8):e0200953. doi: 10.1371/journal.pone.0200953 (PMC6084851; doi:10.1371/journal.pone.0200953)
Supplement: S3 Table — (DOC) [file pone.0200953.s003.doc]

**S3 Table. Correlations between AC**CT subtests and other ET-based tests in healthy controls’ group

|  | d2  *mean latency* | d2  *n°*  *correct* | MCST  *mean latency* | MCST  *n°*  *correct* | MCST  *n° categories* | MCST  *n°*  *cards* | RCPM  *mean latency* | RCPM  *n°*  *correct* | RME  *mean latency* | RME  *n°*  *correct* | RMEc  *mean latency* | RMEc  *n°*  *correct* | IGT  *mean latency* |
| --- | --- | --- | --- | --- | --- | --- | --- | --- | --- | --- | --- | --- | --- |
| ACCT-1 *sd latency*  p-value | .18  .4 | **-.53**  .015 | .12  .6 | -.07  .8 | -.08  .7 | .09  .7 | .08  .7 | -.12  .6 | .006  >.9 | **-.55**  .012 | .34  >.9 | -.18  .4 | .01  >.9 |
| ACCT-2 *sd latency*  p-value | .13  .6 | **-.44**  .047 | -.07  .7 | .21  .4 | .21  .4 | -.19  .4 | **.44**  .046 | -.40  .07 | .18  .4 | .02  >.9 | .37  .09 | -.06  .8 | .28  .2 |
| ACCT-3 *sd latency*  p-value | **.57**  .006 | -.20  .4 | **.55**  .010 | -.40  .07 | -.40  .07 | .32  .2 | **.65**  .002 | -.35  .1 | .07  .8 | .05  .8 | .41  .07 | -.06  .8 | .37  .09 |
| ACCT-4 *sd latency*  p-value | .002  >.9 | -.26  .3 | -.002  >.9 | .03  .9 | .06  .8 | -.22  .4 | -.20  .4 | -.21  .4 | -.001  >.9 | .20  .4 | .13  .6 | .01  >.9 | .20  .4 |
| ACCT-1  *n° correct*  p-value | **-.48**  .027 | .37  .09 | **-.46**  .038 | **.54**  .012 | **.50**  .019 | -.33  .1 | -.21  .4 | **.46**  .034 | .09  .7 | .39  .09 | -.24  .3 | .41  .06 | -.32  .2 |
| ACCT-2  *n° correct*  p-value | .37  .1 | -.26  .3 | .35  .1 | -.39  .08 | -.39  .08 | .43  .053 | .42  .06 | 0  >.9 | .03  .9 | -.23  .3 | .10  .7 | -.17  .5 | -.03  .9 |
| ACCT-3  *n° correct*  p-value | -.38  .09 | .001  >.9 | -.19  .4 | .08  .7 | .10  .7 | -.19  .4 | -.18  .4 | **.44**  .046 | -.16  .5 | .32  .2 | **-.50**  .021 | .28  .2 | -.12  .6 |
| ACCT-4  *n° correct*  p-value | .16  .5 | .23  .3 | .07  .8 | -.01  >.9 | 0  >.9 | .14  .5 | .09  .7 | .19  .4 | -.01  >.9 | -.13  .6 | -.03  .9 | -.15  .5 | -.07  .8 |

Bold numbers indicate statistical significance with *p* < 0.05.

Abbreviations: MCST= Modified Card Sorting test; RCPM= Raven’s Colored Progressive Matrices; RME= Reading the Mind in the eyes test; RMEc= Reading the Mind in the eyes test – control test; IGT= Iowa Gambling Task; sd latency= mean latency standard deviation; n° correct= number of correct responses.
